# Volume of the crocodilian brain and endocast during ontogeny

**DOI:** 10.1371/journal.pone.0178491

**Published:** 2017-06-14

**Authors:** Daniel Jirak, Jiri Janacek

**Affiliations:** 1 MR Unit, Department of Diagnostic and Interventional Radiology, Institute for Clinical and Experimental Medicine, Prague, Czech Republic; 2 Institute of Biophysics and Informatics, 1^st^ Medicine Faculty, Charles University, Prague, Czech Republic; 3 Department of Biomathematics, Institute of Physiology, Czech Academy of Sciences, Prague, Czech Republic; Laboratoire de Biologie du Développement de Villefranche-sur-Mer, FRANCE

## Abstract

Understanding complex situations and planning difficult actions require a brain of appropriate size. Animal encephalisation provides an indirect information about these abilities. The brain is entirely composed of soft tissue and, as such, rarely fossilises. As a consequence, the brain proportions and morphology of some extinct vertebrates are usually only inferred from their neurocranial endocasts. However, because the morphological configuration of the brain is not fully reflected in the endocast, knowledge of the brain/endocast relationship is essential (especially the ratio of brain volume to endocast volume or the equivalent proportion of interstitial tissue) for studying the endocasts of extinct animals. Here we assess the encephalic volume and structure of modern crocodilians. The results we obtained using ex vivo magnetic resonance imaging reveal how the endoneurocranial cavity and brain compartments of crocodilians change configuration during ontogeny. We conclude that the endocasts of adult crocodilians are elongated and expanded while their brains are more linearly organised. The highest proportion of brain tissue to endocast volume is in the prosencephalon at over 50% in all but the largest animals, whereas the proportion in other brain segments is under 50% in all but the smallest animals and embryos. Our results may enrich the field of palaeontological study by offering more precise phylogenetic interpretations of the neuroanatomic characteristics of extinct vertebrates at various ontogenetic stages.

## Introduction

Although the correlation between brain size and behavioural complexity remains ambiguous, brain size, brain dimension compartmentalisation and endoneurocranial space are widely used as measures of behavioural complexity and sensory-locomotive capacity in vertebrates [1]. However, in the case of extinct animals, it is difficult to determine these characteristics because the brain is entirely composed of soft tissue and, therefore, the chances of fossilisation are extremely rare [2]. Hence, the brain proportions and morphology of some extinct vertebrates are usually inferred from their endocasts only [3–4]. The whole enlargement of the brain and the relative dimensional increases in its major components reflect the functional importance of the organ [5]. However, the absence of preserved neural tissues in fossils means that the volume and parts of the brain in extinct animals cannot be calculated accurately enough.

We assess the brain and endocast volume of modern crocodilians in order to explore how the endocast and brain change configuration during ontogeny and to what extent these changes differ in adults of two genera and different size classes. We have chosen crocodilians because (1) they form one of the two remaining extant clades of archosaurs [6] that survived the extinction of non-avian dinosaurs at the end of the Cretaceous era and (2) the rate of their evolution is very slow. Crocodilians, which consist of only 23 extant species, have so perfectly adapted to the lifestyle of amphibious predators that they have been able to survive without major changes for more than 80 million years. The endocasts of most dinosaurs resemble those of extant crocodilians [3]. For this reason, crocodilians serve as a modern analogue for studying ontogenetic changes in the brains of extinct Archosauria. The aim of this study was to obtain imaging data of crocodilian brains and endocasts at various stages of development as a means of replicating a more accurate quantitative analysis of the development of the brain and endocast volume of most extinct dinosaurs.

## Materials and methods

All animal experimentation protocols were approved by the Ethics Committee of the Institute for Clinical and Experimental Medicine and the Ministry of Health of the Czech Republic in accordance with European Communities Council Directive 86/609/EEC. The severed heads of five crocodilians at various stages of development were fixed in 10% formalin for two months. We examined samples of 55 day-old embryos (stage 28 [7]), early and late juveniles, and adults using magnetic resonance imaging (MRI).

Ex vivo MRI of crocodilian heads was performed using a clinical 3-T (three largest specimens) or an experimental 4.7-T scanner (two smallest specimens). These specimens were sealed in airtight plastic bags during MRI scanning to prevent drying and shrinkage [1,8]. The smallest specimen was placed in a tube filled with 4% formalin. Radiofrequency coils were selected according to specimen size. Spin echo- or gradient echo-based MRI sequences were chosen and optimised in order to achieve high resolution and contrast to distinguish different brain structures. The signal-to-noise ratio (SNR) was calculated using SNR = 0.655∙S/σ, where S is signal intensity in the region of interest (ROI), σ is the standard deviation of background noise and constant 0.655 reflects the Rician distribution of background noise in a magnitude MR image. The contrast-to-noise ratio (CNR) was calculated as the difference in SNR between brain and muscle tissue. For volume analysis based on manual segmentation, the following MR sequences were selected using optimised parameters:

*Crocodylus niloticus* and *Caiman crocodilus*—adults: 3-T, head coil, 3D gradient echo sequence (Fast Low Angle SHot, FLASH): Repetition time (TR) = 20 ms, Echo time (TE) = 7 ms, Flip angle = 25°, Resolution = 0.75x0.75x1.00 mm^3^;

*Caiman crocodilus*—late juvenile: 3-T, knee coil, 3D gradient echo sequence FLASH: TR = 20 ms, TE = 8.9 ms, Flip angle = 25°, Resolution = 0.31x0.31x0.30 mm^3^;

*Caiman crocodilus*—early juvenile: 4.7-T, resonator coil, 3D turbo spin echo sequence (Rapid Acquisition with Relaxation Enhancement, RARE): TR = 500 ms, TE = 20 ms, Resolution = 0.20x0.20x0.20 mm^3^.

*Crocodylus acutus*–embryo: 4.7-T, head surface coil, 2D turbo spin echo sequence (Rapid Acquisition with Relaxation Enhancement, RARE): TR = 5500 ms, TE = 32 ms, Resolution = 0.07x0.0.07x0.4 mm^3^.

Table 1 summarises information about the species and the basic imaging parameters used for volumetric analysis.

**Table 1:** Information about specimens and imaging parameters.

	Embryo	Early juvenile	Late juvenile	Adult	Larger adult
**Species**	*Crocodylus acutus*	*Caiman crocodilus*	*Caiman crocodilus*	*Caiman crocodilus*	*Crocodylus niloticus*
**Age**	Stage 28	2 years	3 years	7 years	16 years
**Sex**	Unknown	Female	Female	Male	Female
**MR scanner**	Experimental 4.7-T	Experimental 4.7-T	Clinical 3-T	Clinical 3-T	Clinical 3-T
**MR sequence**	2D turbo spin echo sequence	3D turbo spin echo sequence	3D gradient echo sequence	3D gradient echo sequence	3D gradient echo sequence
**TR [ms]**	5500	500	20	20	20
**TE [ms]**	32	20	8.9	7	7
**Resolution [mm**^**3]**^	0.07x0.0.07x0.40	0.20x0.20x0.20	0.31x0.31x0.30	0.75x0.75x1.00	0.75x0.75x1.00
**Radiofrequency coil**	Head surface coil	Resonator coil	Resonator coil	Head coil	Head coil

We quantified the ratio of endocast to brain volume (EV-BV) only, partial brain volume to whole brain volume indices (pBV-BV), and partial brain volume to partial endocast volume (pBV-pEV) for all samples. We divided the endocast into four major sectors (olfactory (Olf), prosencephalic (Pros), mesencephalic (Mes) and rhombencephalic (Rhomb)). Quantification was based on manual delineation of the region of interest (ROI) using VGStudio MAX software, brain border was in the narrowest part of foramen magnum. ROIs were processed by morphological operations (opening and closing) and then resampled. Extremal surfaces (nasal and foramen magnum) as well as interfaces between brain sectors were delineated by the 3D lasso tool using the Volume Edit module on Amira (FEI) software. Interfaces between prosencephalic and olfactory sectors were set at the front end of the telencephalon convex surface, while interfaces delineating the mesencephalon were set at boundaries of the optical lobe. Resulting volumes were calculated by voxel counting using the Amira module, Material Properties. External surfaces of the brain sectors were obtained from the triangulated surface using the Amira module, Surface Area.

## Results

**Fig 1:**
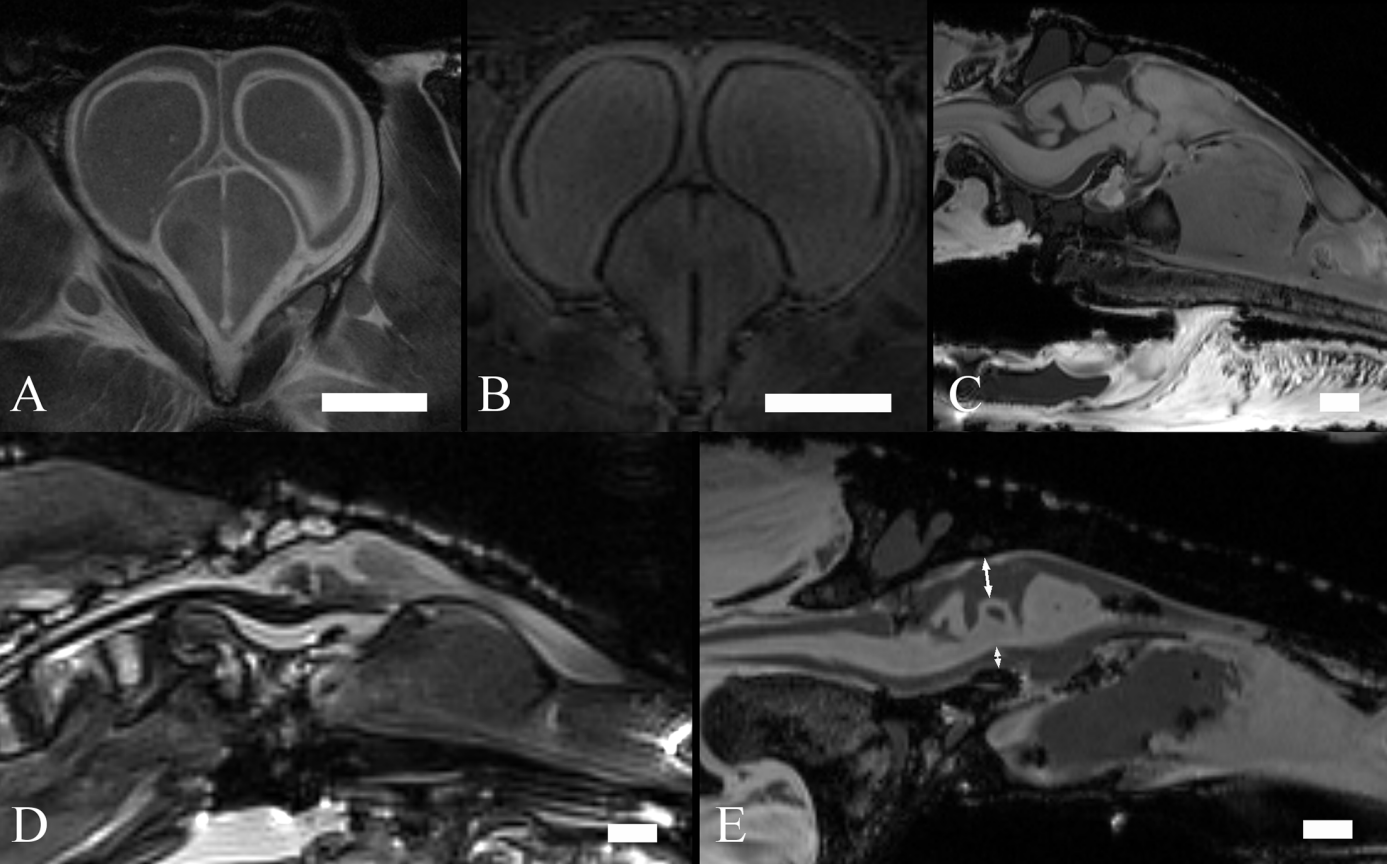
MR images of crocodilian brains. T2w coronal image of an embryo—scan time 3 h 21m (A), T1w coronal image of an early juvenile—scan time 9 h 6 m (B), T1w sagittal image of a late juvenile—scan time 2 h 36 m (C), T2w sagittal image of an adult—scan time 7 m (D), T1w sagittal image of an adult—scan time 40 m (E). Scale bar– 5 mm (A, B, C) and 10 mm (D, E). Double-headed arrows in (E) show the typical area of the interstitium.

Long scan times allowed us to achieve a high signal-to-noise ratio (SNR) and contrast-to-noise ratio (CNR). In the brain tissues of all samples, SNRs varied between 27 and 97, while CNRs between brain tissue and muscles were between 12 and 25. Examples of brain MR imaging of crocodilians at various stages are shown in Fig 1.

The brain volume of the 55-day-old embryo (Fig 1A) was 578 mm^3^ and brain surface was 566 mm^2^. In spite of the high SNR, it was difficult to distinguish brain tissue from other tissues or bones in the embryo. For example, the endocast was practically the same with a volume of 593 mm^3^ and a surface of 572 mm^2^. At later stages of development the endocast was much more distinguishable from brain tissue. The brain volume of the early juvenile caiman (Fig 1B) was 1717 mm^3^ (surface was 1013 mm^2^) and endocast volume was 1974 mm^3^ (surface was 1100 mm^2^). In the case of the late juvenile caiman (Fig 1C), brain volume was 2388 mm^3^ (surface was 1397 mm^2^) while the volume of the brain and the interstitium was 4403 mm^3^ (surface was 1995 mm^2^). In the case of the smaller crocodilian adult (caiman–Fig 1D), brain volume was 5648 mm^3^ (surface was 2568 mm^2^) while the volume of the brain and the interstitium was 11874 mm^3^ (surface was 3871 mm^2^). The brain volume of the largest specimen (crocodile–Fig 1E) was 8538 mm^3^ (surface was 3894 mm^2^) while the volume of the brain and the interstitium was 28984 mm^3^ (surface was 6993 mm^2^). Data are summarised in Table 2.

**Table 2:** Total surface and volume of the brain, interstitium and endocast with EV-BV at various ontogenetic stages.

Stadium	Embryo	Early juvenile	Late juvenile	Adult	Larger adult
Brain surface [mm^2^]	566	1013	1397	2568	3894
Brain volume [mm^3^]	578	1717	2388	5648	8538
Endocast surface [mm^2^]	572	1100	1995	3871	6993
Endocast volume [mm^3^]	593	1974	4403	11874	28984
Interstitium volume [mm^3^]	15	257	2015	6226	20446
EV-BV	1.03	1.15	S1.84	2.10	3.39

Total surface and volume of the brain, interstitium and endocast with EV-BV of embryos (*Crocodylus acutus*), juveniles (*Caiman crocodilus*) and adults (*Caiman crocodilus* and larger *Crocodylus niloticus*)

**Fig 2:**
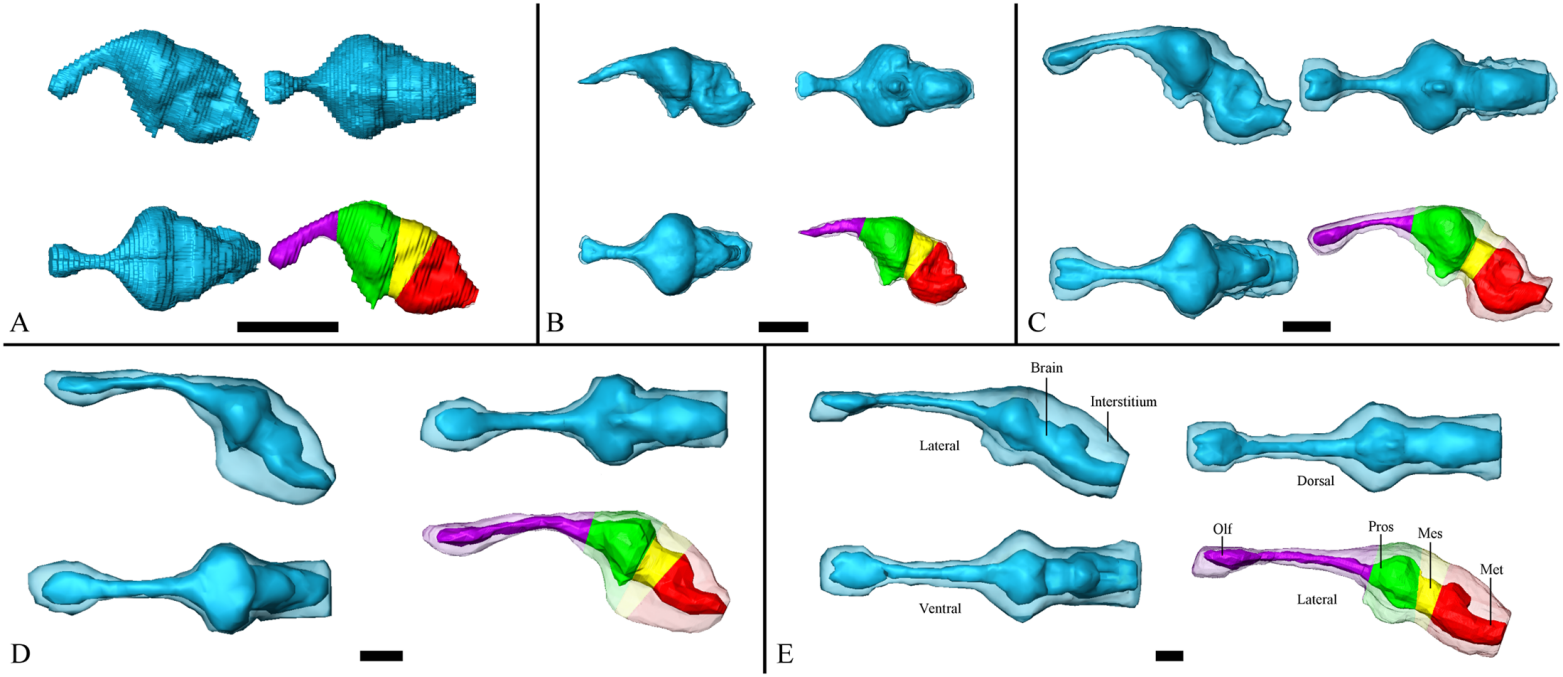
3D reconstructions of the brains and endocasts of crocodilians at various ontogenetic stages divided into four major brain sectors. 3D reconstructions of an embryo (A), early juvenile (B), late juvenile (C), adult (D) and larger adult (E) from ventral (bottom left), lateral (upper left), dorsal (upper right) and lateral (bottom right—major brain sectors coloured) perspectives for each specimen. Brain—dark blue; endocast—light blue. Major brain sectors: olfactory (Olf)—violet, prosencephalic (Pros)—green, mesencephalic (Mes)—yellow and rhombencephalic (Rhomb)—red. Scale bar— 10 mm.

3D reconstructions of the brain and endocast are shown in Fig 2. In *Caiman crocodilus* (Fig 2B–2D), we observed a significant alternation of overall configuration in both the endocast and brain when comparing the early juvenile, late juvenile and adult. This finding suggests that the endocast elongates and expands interstitially and that the brain becomes more linearly organised during ontogeny. This trend is unambiguously supported by data obtained from the embryo, where an S-shaped configuration of the brain was observed (Fig 2A). In contrast, the most linear configuration is evident in the much larger adult species *Crocodylus niloticus* (Fig 2E). The more mature specimens, which have a more linearly organised brain structure, showed higher EV-BV than that of immature specimens.

We also calculated the pBV-BV and pBV-pEV of the four major brain sectors (olfactory (Olf), prosencephalic (Pros), mesencephalic (Mes) and rhombencephalic (Rhomb)) in all samples (Fig 2). The calculation results for the EV-BV, pBV-BV and pBV-pEV of the four major sectors are summarised in Tables 2–4. The prosencephalic sector had the biggest proportion of volume until adulthood, at which stage the rhombencephalon reached a similar pBV-BV value (Table 3). The olfactory sector was the region with the largest relative volume increase among young and adult specimens. The pBV-pEVs in all sectors were close to 1 in the embryonic stage and steadily decreased during development (Table 4). The prosencephalon had the largest pBV-pEV for all intermediate stages among embryos and large adults (Table 4). The proportion of brain tissue to endocast volume in the prosencephalon remained larger than 50% in all except for larger adults, while the proportion in other brain segments was under 50% in all except for early juveniles and embryos (Table 4).

**Table 3:** pBV-BV at various ontogenetic stages.

Sectors	Olf	Pros	Mes	Rhomb
Embryo	0.05	0.49	0.20	0.25
Early juvenile	0.04	0.59	0.13	0.23
Late juvenile	0.06	0.54	0.09	0.31
Smaller adult	0.12	0.51	0.14	0.22
Larger adult	0.16	0.36	0.11	0.37

Calculated partial/total volume (pBV-BV) of the brains of embryos (*Crocodylus acutus*), juveniles (*Caiman crocodilus*) and adults (*Caiman crocodilus* and larger *Crocodylus niloticus*). Sectors: Olf—olfactory, Pros—prosencephalic, Mes—mesencephalic and Rhomb—rhombencephalic

**Table 4:** pBV-pEV at various ontogenetic stages.

Sectors	Olf	Pros	Mes	Rhomb
Embryo	0.99	0.97	0.98	0.96
Early juvenile	0.66	0.91	0.87	0.81
Late juvenile	0.28	0.71	0.46	0.46
Smaller adult	0.34	0.66	0.43	0.35
Larger adult	0.25	0.32	0.26	0.30

Calculated ratios of partial brain volume to partial endocast volume (pBV-pEV) for early embryos (Crocodylus acutus), juveniles (*Caiman crocodilus*) and adults (*Caiman crocodilus* and larger *Crocodylus niloticus*) divided into four major sectors: Olf—olfactory, Pros—prosencephalic, Mes—mesencephalic and Rhomb—rhombencephalic

## Discussion

In non-avian reptiles, brain volume is traditionally estimated using brain mass, where there is usually a high correlation between both parameters with the endocast volume ratio roughly estimated at 0.5 [9]. The novel approach of using laser scan data has been employed to analyse the relative sizes of brains and cerebra of several theropods in comparison with non-avian reptiles [10]. In our study, we used MRI for brain and interstitium visualisation because MRI is a superior method for soft tissue imaging [11–12]. All specimens were fixed in formalin. We demonstrated that post-mortem subject volume variation was significantly reduced across time points relative to inter-subject volume variation over a period of one week to six months [13]. We also concluded that ex vivo/in vivo brain volumetrics are linearly correlated and, therefore, ex vivo MR volumetry can accurately capture the ante-mortem endoneurocranial anatomy [13]. We consider it likely, therefore, that shrinkage was insignificant compared to methodological errors caused by manual segmentation in our study.

Results obtained from MR images of crocodilian heads revealed that the brain occupied 29% of endocast volume in the largest crocodile (Table 2). A similar percentage was achieved (32%) using a different method of brain volume assessment for the largest alligator [14], where the brain mass to endocast volume ratio was calculated; brain weight included the pia mater but excluded the pituitary gland, dura mater, arachnoid, grossly visible blood vessels, olfactory tracts and any dried blood. We also observed that endocast volume increased faster than brain mass relative to total length in alligators. Our results are in agreement with the premise that increased endocast size affects EV-BV ratio. The high pBV-EV in the prosencephalon and rhombencephalon reflected the importance of the forebrain in cognition and behaviour and of the cerebellum in mobility coordination.

Although the more linear adult configuration is an ontogenetically-related phenomenon, it is the only available analogue for explaining the similar evolutionary transformation of the endocast during the period in which gigantic forms of extinct vertebrates first emerged. The endocasts of non-avian dinosaurs strongly resemble those of crocodilians both in general proportions and specific anatomic features [14]. Moreover, MR data show that in adults the linear-shaped endoneurocranium encases a similarly linearly-arranged brain quite uniformly enveloped by a thicker interstitium. While brain volume in caimans increased 3.5-fold, endoneurocranial volume enlarged 6.1-fold. Thus, the larger endoneurocranium, which is ontogenetically more mature, provides less information about the shape of the brain. Modern crocodilians and birds are the only living groups descended from the Archosauria, to which dinosaurs also belong. However, as opposed to crocodilians, the endoneurocrania of birds do not exhibit increased interstitial volume [15]. When placed in the context of the highly conservative form of crocodilian evolution, these facts support the hypothesis that crocodilians serve as the only relevant extant model of extinct dinosaurs for studying the ontogenetic development of the brain and endoneurocranium. Several studies have revealed notable differences in the endocast configurations of tyrannosaurs of various sizes [16–18]. The anterior parts of dinosaur endocasts have been shown to mould the developing braincase walls with the mid- and hind-brain enveloped by extensive blood and lymphatic sinuses [2]. This might imply that the pattern of EV-BV observed in modern crocodilians of conservative evolution is typical of large archosaurs. Therefore, the modern crocodilian model may improve phylogenetic interpretations of the neuroanatomic characteristics of some extinct vertebrates and also contribute to explaining the evolutionary development of the central nervous system at various ontogenetic stages.

Our results may also support the hypothesis that a similar transformation in the evolutionary patterns of endocasts also occurred in extinct vertebrates. This may in turn explain why in evolutionary younger gigantic tyrannosaurs, the linear-type endocast is observed in comparison to small and much older tyrannosaurids [19]. Therefore, the encephalic volumes of large extinct vertebrates would probably have been much smaller compared to their endocasts. The first clue towards determining how much smaller the brain might have been is to calculate the EV-BV in modern crocodilians. In evolutionary younger dinosaurs such as the species *Tyrannosaurus rex*, we recommend an EV-BV of around 3.4 for deriving brain volume from endocast volume. But in the case of the earlier and most primitive members of the Tyrannosauroidea, e.g. the species *Dilong paradoxus* [20], our data suggest that an EV-BV of slightly above 1 should be used for calculating soft brain tissue. We also expect there would have been a high pBV-pEV value for the forebrain in earlier ontogenetic phases and higher pBV-pEV values for the forebrain and hindbrain over the whole duration of ontogenesis.

## Conclusions

We demonstrate that the endocast is elongated and expanded, while the brain is more linearly organised and almost uniformly enveloped by interstitial tissue in larger and more mature modern crocodilians. The highest proportion of brain tissue to endocast volume is in the prosencephalon, followed by the rhombencephalon. Modern crocodilians of conservative evolution serve as an excellent model for analysing brain development in most dinosaurs. Therefore, the results of this study may enrich the field of palaeontological study by providing more accurate phylogenetic interpretations of the neuroanatomic characteristics of extinct vertebrates at various ontogenetic stages.

## Supporting information

S1 Fig 3D visualization of crocodilian embryo brain. 3D image of crocodilian embryo brain in vrml format.
(WRL)

S2 Fig 3D visualization of crocodilian embryo endocast. 3D image of crocodilian embryo endocast in vrml format.
(WRL)

S3 Fig 3D visualization of crocodilian early juvenile brain. 3D image of crocodilian early juvenile brain in vrml format.
(WRL)

S4 Fig 3D visualization of crocodilian early juvenile endocast. 3D image of crocodilian early juvenile endocast in vrml format.
(WRL)

S5 Fig 3D visualization of crocodilian late juvenile brain. 3D image of crocodilian late juvenile brain in vrml format.
(WRL)

S6 Fig 3D visualization of crocodilian late juvenile endocast. 3D image of crocodilian late juvenile endocast in vrml format.
(WRL)

S7 Fig 3D visualization of crocodilian adult brain. 3D image of crocodilian adult brain in vrml format.
(WRL)

S8 Fig 3D visualization of crocodilian adult endocast. 3D image of crocodilian adult endocast in vrml format.
(WRL)

S9 Fig 3D visualization of crocodilian large adult brain. 3D image of crocodilian large adult brain in vrml format.
(WRL)

S10 Fig 3D visualization of crocodilian large adult endocast. 3D image of crocodilian large adult endocast in vrml format.
(WRL)
